# Visual Discrimination, Serial Reversal, and Extinction Learning in the *mdx* Mouse

**DOI:** 10.3389/fnbeh.2019.00200

**Published:** 2019-08-30

**Authors:** Price E. Dickson, Guy Mittleman

**Affiliations:** ^1^The Jackson Laboratory, Bar Harbor, ME, United States; ^2^Department of Psychological Science, Ball State University, Muncie, IN, United States

**Keywords:** Duchenne muscular dystrophy, dystrophin, C57BL/10ScSn-Dmd^*mdx*^, operant conditioning, touchscreen, behavioral flexibility, cognitive flexibility, stimulus salience

## Abstract

Duchenne muscular dystrophy (DMD) is the most common form of muscular dystrophy and the most common neuromuscular disorder. In addition to neuromuscular consequences, some individuals with DMD experience global intellectual dysfunction and executive dysfunction of unknown mechanistic origin. The cognitive profile of the *mdx* mouse, the most commonly used mouse model of DMD, has been incompletely characterized and has never been assessed using the touchscreen operant conditioning paradigm. The touchscreen paradigm allows the use of protocols that are virtually identical to those used in human cognitive testing and may, therefore, provide the most translational paradigm for quantifying mouse cognitive function. In the present study, we used the touchscreen paradigm to assess the effects of the *mdx* mutation on visual discrimination learning, serial reversal learning, and extinction learning. To enable measuring task-dependent learning and memory processes while holding demands on sensory-driven information processing constant, we developed equally salient visual stimuli and used them on all experimental stages. Acquisition of the initial pairwise visual discrimination was facilitated in *mdx* mice relative to wildtype littermates; this effect was not explained by genotypic differences in impulsivity, motivation, or motor deficits. The *mdx* mutation had no effect on serial reversal or extinction learning. Together, findings from this study and previous studies suggest that *mdx* effects on cognitive function are task-specific and may be influenced by discrimination type (spatial, visual), reward type (food, escape from a non-preferred environment), sex, and genetic background.

## Introduction

Duchenne muscular dystrophy (DMD) is a recessive X-linked neuromuscular disease affecting 1 in 3,500 human males; it is the most common form of muscular dystrophy and the most common neuromuscular disorder (Hoffman and Kunkel, 1989; Willmann et al., 2009). DMD is caused by a mutation in the DMD gene, the largest gene in the human genome. The *DMD* mutation results in an absence of functional dystrophin, a cytoskeletal protein that is a critical component of the dystrophin–glycoprotein complex (Ervasti, 2007). The absence of functional dystrophin results in progressive muscle degeneration and weakness which is ultimately fatal due to heart failure in the second or third decade of life.

In addition to cardiac and skeletal muscles, dystrophin is expressed in the cerebellum, the cerebral cortex, and the hippocampus (Lidov et al., 1990; Huard and Tremblay, 1992). The absence of functional dystrophin in these brain regions is believed to underlie the cognitive deficits that are present in a subset of individuals with DMD (Cyrulnik and Hinton, 2008). These deficits include global intellectual dysfunction as well as executive dysfunction (e.g., cognitive flexibility and working memory; Snow et al., 2013). There is remarkable heterogeneity in the type and degree of cognitive deficits in this population. However, the mechanisms through which dystrophin dysfunction drives these deficits are largely unknown.

The *mdx* mouse (Bulfield et al., 1984) is the most commonly used mouse model of DMD and lacks functional dystrophin in muscle tissue and brain due to a spontaneous mutation in exon 23 of the *Dmd* gene. Abnormalities in brain structure, biochemistry, and neurophysiological function of mice lacking functional dystrophin have been observed and include changes in cellular antioxidant defenses, osmoregulation, neurotransmission, and synaptic plasticity (Vaillend et al., 1999, 2004; Vaillend and Billard, 2002; Dallerac et al., 2011; Cohen et al., 2015; Xu et al., 2015; Vaillend and Chaussenot, 2017; Pereira da Silva et al., 2018); these biological effects may underlie the cognitive deficits associated with a lack of functional dystrophin. Although pathophysiology of the *mdx* mouse has been studied extensively (Manning and O’Malley, 2015), the cognitive profile of the *mdx* mouse has been studied much less so. In particular, the effects of dystrophin perturbation on cognitive flexibility remain largely unexplored. In that regard, cognitive functions of the *mdx* mouse including cognitive flexibility have never been assessed using the touchscreen operant conditioning paradigm (Izquierdo et al., 2006; Brigman and Rothblat, 2008; Brigman et al., 2009; Dickson et al., 2013, 2014, 2017). The touchscreen paradigm allows the use of protocols which are virtually identical to those used in human cognitive testing such as the Cambridge Neuropsychological Test Automated Battery (Robbins et al., 1994). Therefore, touchscreen paradigms in mice may provide the most translational paradigm for modeling the effects of dystrophin perturbation on cognitive function.

In the present study, we used a touchscreen operant conditioning paradigm similar to those we (Dickson et al., 2013, 2014, 2017) and others (Izquierdo et al., 2006; Brigman and Rothblat, 2008; Brigman et al., 2009) have used previously to assess the effects of the *mdx* mutation on visual discrimination learning, serial reversal learning, and extinction learning. In addition to cognitive performance on each of these learning stages, we considered variables including response propensity and latency as well as reward collection propensity and latency to dissociate potential effects of the *mdx* mutation on learning and cognitive flexibility from effects on neuromuscular function, impulsivity, and reward valence. We hypothesized that the *mdx* mutation would impair learning performance at one or more stages of the touchscreen assay.

## Materials and Methods

### Subjects

The following experiments were approved by the Institutional Animal Care and Use Committee at the University of Memphis and conducted in accordance with the National Institutes of Health Guidelines for the Care and Use of Laboratory Animals. Efforts were made to reduce the number of animals used and to minimize animal pain and discomfort.

Male mice hemizygous for the *Dmd^mdx^* spontaneous mutation (C57BL/10ScSn-*Dmd^mdx^*/J, #001801) and female wildtype mice with the same genetic background (C57BL/10ScSnJ, #000476) were purchased from The Jackson Laboratory (Bar Harbor, ME, USA). Two phases of breeding were required to produce experimental mice. In the first phase, hemizygous male mice were mated with wildtype female mice to produce litters of heterozygous females and wildtype males. In the second phase, heterozygous female mice were mated with wildtype male mice to produce litters containing both hemizygous and wildtype males. These hemizygous male mice and their male littermate controls were used as experimental subjects. This breeding protocol was chosen because it produced litters consisting of wildtype and hemizygous experimental mice at a 1:1 ratio. We used littermates in order to control for litter effects that could be driven by differences in maternal behavior and intrauterine environment. All breeding cages contained a single male and single female to enable correct identification of litter. Genotyping of all mice used in the study was performed by Transnetyx (Cordova, TN, USA).

We tested *mdx* mice (*n* = 23) and wildtype littermates (*n* = 23) from 23 litters. By sampling a single hemizygote and a single littermate control from each litter, we controlled for the possibility that a single litter would exert a disproportionate effect on group means. Mice were continuously maintained in a temperature-controlled environment (21 ± 1°C) on a 12:12 light:dark cycle. After weaning at 4 weeks of age, experimental subjects were housed in groups of 3–5 until they entered the experiment; mice were then individually housed to facilitate food restriction. Mice had free access to food until they were individually housed, at which point they were food-restricted such that body weight was 90% of baseline weight at the beginning of each daily operant conditioning session. Mice always had free access to water in the home cage. Food restriction is used in most operant conditioning studies that rely on food to positively reinforce a lever press or nosepoke response (e.g., Izquierdo et al., 2006; Brigman and Rothblat, 2008; Brigman et al., 2009; Dickson et al., 2013, 2014, 2017).

### Apparatus

Behavioral training and testing were conducted in operant conditioning chambers (Lafayette Instruments, Lafayette, IN, USA; Med Associates, St. Albans, VT, USA) which have been described in detail previously (Dickson et al., 2013). Briefly, the front wall of each chamber consisted of an infrared touchscreen. The rear wall consisted of: (1) a centrally mounted liquid dipper which provided access to 0.01 cc of Silk Vanilla Soymilk as a reward; (2) a trial initiation stimulus light located above the food receptacle; and (3) a house light centrally mounted at the top of the chamber. Operant conditioning chambers were controlled by a Lafayette Instruments control unit running ABET II and Whisker software. All operant conditioning schedules were written in-house using ABET II.

### Operant Conditioning: Pairwise Visual Discrimination, Serial Reversals, and Extinction

Mice began the experiment at 12 weeks of age and were trained and tested in the same chamber and at the same time daily 7 days per week until they completed the experiment. Behavioral training and testing were conducted using methods similar to those previously described (Dickson et al., 2013).

#### Training Mice to Use the Operant Conditioning Chambers

Prior to testing, mice were trained to: (1) nosepoke a stimulus displayed on the touchscreen at the front of the chamber; (2) collect a food reward at the back of the chamber following the nosepoke to the touchscreen; and (3) initiate the next trial by making a nosepoke to the food receptacle at the back of the chamber. Following training, mice were tested on a pairwise visual discrimination, four serial reversals of that discrimination, and 26 extinction sessions.

#### Visual Stimuli

**Figure 1:**
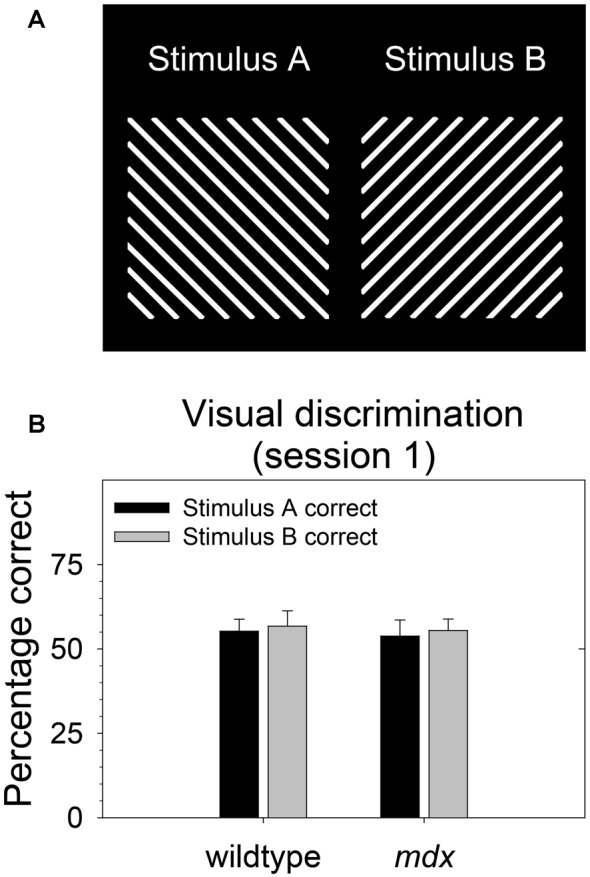
Stimulus salience. Visual stimuli used in the experiment were equally salient. **(A)** During all stages of the experiment, mice (23 *mdx* and 23 wildtype littermates) discriminated a single pair of unidimensional visual stimuli presented on the touchscreen. Rewarded stimulus was counterbalanced. **(B)** We quantified salience of the two stimuli used in the study by assessing the relative preference for each stimulus on the first session of the acquisition of the pairwise visual discrimination. There was no significant main effect of rewarded stimulus, genotype, or their interaction (*p* > 0.70 for all tests) indicating that the two stimuli were equally salient for both wildtype and *mdx* mice. Moreover, there was no effect of rewarded stimulus on any stage in the study.

During behavioral testing, mice discriminated a single pair of unidimensional visual stimuli presented on the touchscreen (Figure 1A). Stimuli were 6.5 cm wide and 6.5 cm high. Stimulus A was rotated 90° to create stimulus B. Thus, the two stimuli were identical in all respects (e.g., size, brightness) except for orientation. In contrast to our previous studies (Dickson et al., 2013, 2014, 2017), the visual stimuli used in the present study were designed to be equally salient. Specifically, stimuli were equally bright, and brightness was equally distributed across stimulus regions. The goal of this was to enable the probing of task-dependent (i.e., top-down) learning and memory processes while holding constant the demands on sensory-driven (i.e., bottom-up) information processing.

#### Pairwise Visual Discrimination

Each session started with the illumination of the house light at the back of the chamber and the beginning of the first trial. At the beginning of each trial, the trial initiation stimulus light at the back of the chamber was illuminated signaling that the mouse could start the trial by making a nosepoke into the food receptacle. When a nosepoke occurred, the stimulus light was turned off and the two visual stimuli were randomly presented on the right and left sides of the touchscreen at the front of the chamber. Stimulus A was correct for 11 of the litters. Stimulus B was correct for 12 of the litters. A nosepoke to the correct stimulus resulted in access to the 0.01 cc vanilla soymilk reward (i.e., liquid dipper was raised and then lowered after 10 s). A nosepoke to the incorrect stimulus resulted in a 10 s timeout which was signaled by turning off the house light. Immediately following a nosepoke to either stimulus, the visual stimuli were removed from the screen. A 5 s intertrial interval (ITI) followed reward or timeout, after which the trial initiation light above the food receptacle was illuminated signaling that the mouse could initiate another trial.

As we (Dickson et al., 2010, 2013, 2014, 2017) and others (Izquierdo et al., 2006; Brigman and Rothblat, 2008; Brigman et al., 2009) have done previously, a correction procedure was used to eliminate the development of a side bias: during each session, the second and all subsequent trials were considered either “correction” or “non-correction” trials depending on the correctness of the previous trial. Specifically, a correction trial followed an incorrect trial and a non-correction trial followed a correct trial. During correction trials, stimulus presentation was not randomized. Rather, the correct and incorrect stimuli were presented on the same side as in the previous trial. The purpose of this was to prevent the mouse from developing a strategy in which the mouse ignored the visual stimuli, always chose the same side and was therefore rewarded on 50% of the trials. A non-correction trial followed a correct trial, and stimulus presentation was randomized.

Sessions continued in this manner until 64 trials were completed or 60 min had elapsed, whichever occurred first. Both correction and non-correction trials were counted towards the 64-trial maximum per session. Mice reached criterion when they completed a single session at 80% correct (calculated using non-correction trials). After reaching criterion on the final session of the visual discrimination stage, mice were advanced to the reversal stage on the following session.

#### Serial Reversals of a Pairwise Visual Discrimination

Mice were tested on a series of four serial reversals. Serial reversal stages were identical to the acquisition stage with the exception that the response contingencies were reversed relative to the previous stage. Specifically, mice that were rewarded for nosepoking stimulus A during the acquisition stage were rewarded for nosepoking stimulus B on reversal 1, stimulus A on reversal 2, stimulus B on reversal 3, and stimulus A on reversal 4. Conversely, mice that were rewarded for nosepoking stimulus B during the acquisition stage were rewarded for nosepoking stimulus A on reversal 1, stimulus B on reversal 2, stimulus A on reversal 3, and stimulus B on reversal 4. On each reversal stage, mice reached criterion when they completed a single session at 80% correct (calculated using non-correction trials). After reaching criterion on reversals one, two, and three, mice were advanced to the next reversal stage on the following session. After reaching criterion on reversal four, mice were advanced to the extinction stage on the following session.

#### Extinction of a Pairwise Visual Discrimination

The extinction stage was identical to reversal four with the exception that correct responses were not rewarded. Specifically, when the mouse nosepoked the correct stimulus, the dipper arm was not raised. Mice were tested for 26 extinction sessions. Following extinction session 26, the experiment was terminated, and mice were immediately returned to a free feeding schedule.

### Dependent Variables

The following dependent variables were collected on each session of the visual discrimination, serial reversal, and extinction stages: number of correct and error responses (non-correction trials only), number of trials completed, latency to stimulus choice for correct and error responses, propensity and latency to collect a reward following a correct response, propensity and latency to attempt to collect a reward following an error response. Latency to stimulus choice was defined as the time in seconds between stimulus onset and a nosepoke to one of the stimuli presented on the screen. Propensity to collect a reward was defined as the percentage of correct trials on which a food receptacle head entry occurred during the 10 s reward following a nosepoke to the correct visual stimulus. Propensity to attempt to collect a reward was defined as the percentage of error trials on which a food receptacle head entry occurred during the 10 s timeout following a nosepoke to the incorrect visual stimulus. Latency to collect a reward was defined as the time in seconds between a nosepoke to the correct stimulus on the screen and a head entry into the food receptacle. Latency to attempt to collect a reward was defined as the time in seconds between a nosepoke to the incorrect stimulus on the screen and a head entry into the food receptacle.

### Statistical Methods

Analysis of variance (ANOVA) was used to assess performance on the visual discrimination, serial reversal learning, and extinction stages. Normality of all measures was assessed by inspecting normal probability plots. The assumption of homogeneity of variance across groups was assessed using Mauchly’s test of sphericity. The Huynh–Feldt correction was used when this assumption was violated. The criterion for statistical significance was *p* < 0.05. When performing multiple comparisons, Fisher’s Least Significant Difference procedure was used.

## Results

### Stimulus Salience

As we and others have done previously (Bussey et al., 2008; Dickson et al., 2013), we quantified salience of the two stimuli used in the study (Figure 1A) by assessing the relative preference for each stimulus on the first session of the acquisition of the pairwise visual discrimination. We performed a 2 × 2 between-subjects ANOVA using percentage correct as the dependent measure, rewarded stimulus (stimulus A, stimulus B) as a between-subjects factor, and genotype (wildtype, *mdx*) as a second between-subjects factor. There was no significant effect of rewarded stimulus, genotype, or their interaction (*p* > 0.70 for all tests) indicating that stimulus A and stimulus B were equally salient for both wildtype and *mdx* mice (Figure 1B). Moreover, first-pass analysis of data from the visual discrimination, serial reversal, and extinction stages indicated that there was no effect of rewarded stimulus on any stage in the study. Therefore, we dropped rewarded stimulus as a factor in subsequent analyses.

### Pairwise Visual Discrimination Learning

**Figure 2:**
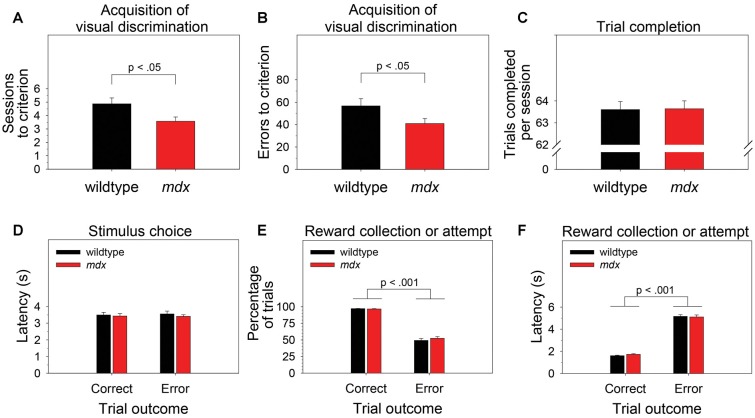
Visual discrimination learning. *mdx* mice (*n* = 23) exhibited facilitated pairwise visual discrimination relative to wildtype littermates (*n* = 23).** (A,B)** Relative to wildtype littermates, *mdx* mice required significantly fewer sessions to reach criterion and committed significantly fewer errors prior to reaching criterion. **(C–F)** These performance differences were not driven by genotype differences in impulsivity, motivation, or motor function: **(C)** *mdx* and wildtype mice completed almost all the available 64 trials per session. **(D)** *mdx* and wildtype mice rapidly nosepoked the correct or incorrect stimulus after the stimuli appeared on the touchscreen, and there was no relationship between latency to make a stimulus choice and trial outcome. **(E)** *mdx* and wildtype mice collected the reward following a correct stimulus choice on almost all trials; they attempted to collect a reward following an incorrect stimulus choice significantly less frequently. **(F)** *mdx* and wildtype mice collected the reward rapidly following a correct stimulus choice; they attempted to collect a reward significantly less rapidly following an incorrect stimulus choice.

To examine the effect of the *mdx* mutation on pairwise visual discrimination learning, we performed one-way ANOVAs using sessions to criterion or errors to criterion as the dependent measure and genotype (wildtype, *mdx*) as a between-subjects factor. Relative to wildtype mice, *mdx* mice required significantly fewer sessions to reach criterion (Figure 2A; *F*_(1,42)_ = 5.77, *p* < 0.05) and committed significantly fewer errors prior to reaching criterion (Figure 2B; *F*_(1,42)_ = 4.10, *p* < 0.05). Genotype explained 11.9% of the variance in sessions to criterion (*η*^2^ = 0.119) and 8.7% of the variance in errors to criterion (*η*^2^ = 0.087).

To test the hypothesis that these performance differences were driven by phenotypes unrelated to learning and memory (e.g., motivation, impulsivity, motor function), we performed one-way or mixed-model ANOVAs to examine the effects of the *mdx* mutation on number of trials completed per session, the latency to choose the correct or incorrect stimulus following trial initiation, the propensity to collect or attempt to collect a reward following a correct or incorrect stimulus choice, respectively, and the latency to do so. Genotype (wildtype, *mdx*) was the between-subjects factor in all ANOVAs. Trial outcome (correct, error) was a within-subjects factor in most ANOVAs.

Briefly, there was no significant effect of genotype on any of these measures. Mice completed almost all the available 64 trials per session (Figure 2C). Mice rapidly nosepoked the correct or incorrect stimulus after the stimuli appeared on the touchscreen, and there was no relationship between latency to make a stimulus choice and trial outcome (i.e., impulsive responding did not underlie incorrect choices; Figure 2D). Mice collected the reward following a correct stimulus choice on almost all trials and attempted to collect a reward following an incorrect stimulus choice significantly less frequently (Figure 2E; *F*_(1,42)_ = 575.14, *p* < 0.001). Mice collected the reward rapidly following a correct stimulus choice and attempted to collect a reward significantly less rapidly following an incorrect stimulus choice (Figure 2F; *F*_(1,42)_ = 944.019, *p* < 0.001).

### Serial Reversal Learning

**Figure 3:**
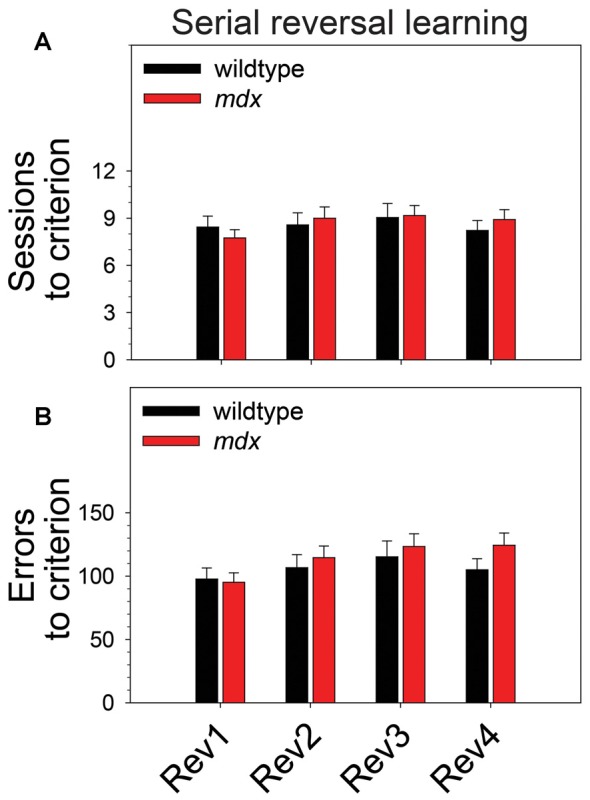
Serial reversal learning. Serial reversal learning of *mdx* mice (*n* = 23) and wildtype littermates (*n* = 23) did not differ. **(A)** There was no main effect of genotype, no main effect of reversal stage, and no interaction of these two factors on sessions to criterion. **(B)** There was a significant main effect of reversal stage on errors to criterion that was driven by a significant increase in the number of errors committed across reversal stages. Specifically, the number of errors committed on the second, third, and fourth reversal stages was significantly greater than the number committed on the first reversal stage (*p* < 0.05 for all comparisons). Although this effect was more robust in *mdx* mice than wildtype littermates, the interaction of genotype and reversal stage was not significant, and there was no significant main effect of genotype.

To examine the effect of the *mdx* mutation on serial reversal learning of a pairwise visual discrimination, we performed repeated-measures ANOVAs using sessions to criterion or errors to criterion as the dependent measure, genotype (wildtype, *mdx*) as a between-subjects factor, and reversal stage (1–4) as a repeated-measures factor. For sessions to criterion (Figure 3A), there was no main effect of genotype, no main effect of reversal stage, and no interaction of these two factors. For errors to criterion (Figure 3B), there was a significant main effect of reversal stage (*F*_(3,132)_ = 4.45, *p* < 0.01). *Post hoc* tests indicated that this effect was driven by a significant increase in the number of errors committed across reversal stages. Specifically, the number of errors committed on the second, third, and fourth reversal stages was significantly greater than the number committed on the first reversal stage (*p* < 0.05 for all comparisons). Although this effect was more robust in *mdx* mice than wildtype littermates, the interaction of genotype and reversal stage was not significant, and there was no significant main effect of genotype.

### Extinction Learning

To examine the effect of the *mdx* mutation on extinction of a pairwise visual discrimination, we performed mixed-model ANOVAs using the following variables as dependent measures: number of trials completed, percentage of correct responses, side bias, latency to make a stimulus choice, attempts to collect a reward following a correct response, and latency to attempt to collect a reward following a correct response. Note that a food reward could not be collected following a correct response on the extinction stage because the liquid dipper was not activated. In all ANOVAs, genotype (wildtype, *mdx*) was the between-subjects factor and extinction session (1–26) was the repeated-measures factor. Analysis of extinction learning was performed using 21 wildtype and 23 *mdx* mice because two wildtypes were not tested on the extinction stage.

**Figure 4:**
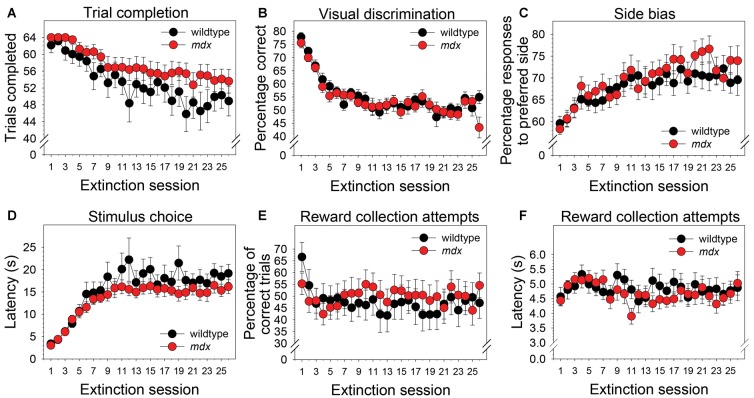
Extinction learning. Extinction learning of *mdx* mice (*n* = 23) and wildtype littermates (*n* = 21) did not differ. Neither a significant main effect of genotype nor a significant interaction of genotype and session were observed for any of the dependent measures collected during the extinction stage. As a group, mice exhibited **(A)** a modest but significant decrease in the number of trials completed across the 26 extinction sessions, **(B)** a significant decrease in percentage correct, **(C)** a significant increase in side bias, **(D)** a significant increase in latency to make a stimulus choice, and **(E)** a significant decrease in the percentage of trials on which mice attempted to collect a reward following a correct response. **(F)** Eleven wildtype mice (52%) and 15 *mdx* mice (65%) attempted to collect a reward on all 26 extinction sessions. For these mice, latency to attempt to collect a reward following a correct response did not change significantly across sessions.

Neither a significant main effect of genotype nor a significant interaction of genotype and session were observed for any of the dependent measures collected during the extinction stage. Across the 26 extinction sessions, mice as a group exhibited a significant decrease in the number of trials completed (Figure 4A; *F*_(25,1050)_ = 11.99, *p* < 0.001), a significant decrease in percentage correct (Figure 4B; *F*_(25,1050)_ = 18.22, *p* < 0.001), a significant increase in side bias (Figure 4C; *F*_(25,1050)_ = 5.85, *p* < 0.001), a significant increase in latency to make a stimulus choice (Figure 4D; *F*_(25,1050)_ = 19.47, *p* < 0.001), and a significant decrease in the percentage of correct trials on which a reward collection attempt was made (Figure 4E; *F*_(25,1050)_ = 2.03, *p* < 0.05). Eleven wildtype mice (52%) and 15 *mdx* mice (65%) made reward collection attempts on all 26 extinction sessions. For these mice, repeated-measures ANOVA indicated that the latency to make a reward collection attempt following a correct response did not change significantly across sessions (Figure 4F; *F*_(25,600)_ = 1.63, *p* = 0.06).

## Discussion

We assessed the effects of the *mdx* mutation on discrimination learning, serial reversal learning (an index of cognitive flexibility), and extinction learning. To accomplish this, *mdx* mice on a C57BL/10ScSnJ background and wildtype littermates were tested on a series of touchscreen-based food-reinforced pairwise visual discriminations that have been adapted from those used with humans and nonhuman primates. To enable measuring task-dependent learning and memory processes while holding demands on sensory-driven information processing constant, we developed visual stimuli that were equally salient (Figure 1) and used these stimuli on all stages of the experiment. Acquisition of the initial pairwise visual discrimination was facilitated in *mdx* mice relative to wildtype littermates (Figures 2A,B); this effect was not explained by genotypic differences in impulsivity, motivation, or motor deficits (Figures 2C–F). During serial reversals, mice performed significantly worse on the final three reversals relative to the first (Figure 3), but there was no effect of the *mdx* mutation on serial reversal learning. During extinction, mice exhibited only a modest reduction in the number of completed trials per session despite correct responses no longer being rewarded (Figure 4A). Despite this high level of responding during extinction sessions, visual discrimination performance rapidly dropped to chance levels (Figure 4B), a significant side bias emerged (Figure 4C), and latency to make a correct response on the touchscreen increased from 3 to 18 s (Figure 4D). There was no effect of the *mdx* mutation on extinction learning.

### Visual Discrimination Learning

In the present study, *mdx* mice exhibited significantly better touchscreen-based pairwise visual discrimination than their wildtype littermates (Figures 2A,B). To determine if this genotypic effect on visual discrimination learning could be better explained by an effect on another cognitive function, we compared performance of *mdx* and wildtype littermates on several other task-related variables. First, we reasoned that an effect of the *mdx* mutation on impulsivity would be revealed by genotype group differences in latency (beginning from trial initiation) to make a nosepoke to the correct or incorrect visual stimulus on the touchscreen. Second, we reasoned that differences in reward valence, motivation to perform the task, or ability to perform the task would be revealed by genotype group differences in latency to collect a reward, number of rewards collected, or number of trials completed. Finally, we reasoned that an increase in the number of attempted reward collections following an incorrect nosepoke to the touchscreen would reveal uncertainty about the correctness of the response driven by impaired ability to use visual feedback (i.e., house light turning off) or auditory feedback (absence of the motor raising the dipper arm). We observed no differences between the *mdx* mice and wildtype littermates on any of these indexes (Figures 2C–F). Collectively, these data indicate that the facilitated visual discrimination in *mdx* mice was independent of effects of the *mdx* mutation on impulsivity, reward valence, motivation to perform the task, ability to perform the task, or ability to use visual or auditory feedback.

Our observation from the current study that *mdx* mice exhibit facilitated visual discrimination relative to littermate controls is consistent with a previous study by Lewon et al. (2017) in which *mdx* mice outperformed wildtype littermates on an operant food-reinforced lever-press based spatial discrimination. In contrast, Vaillend et al. (1995) found that mdx mice did not differ from controls in the acquisition of a food reinforced lever-pressing task. Studies in which learning in *mdx* mice has been assessed using maze-based assays (e.g., Barnes maze, Morris water maze, radial arm maze, etc.) have observed either no difference in acquisition between *mdx* and control mice (Sesay et al., 1996; Vaillend et al., 1998; Remmelink et al., 2016), an impairment in *mdx* mice (Chaussenot et al., 2015), or a more complex phenotype (Vaillend et al., 2004).

Core differences between operant conditioning and non-operant conditioning protocols may underlie observed differences between these two categories of studies. For example, food restriction and food reinforcement are used in operant conditioning paradigms whereas no food restriction and escape from water as a motivator are used in water maze paradigms. It is also worth considering that most of the studies cited here have assessed the performance of male mice, although the performance of females has been described (Remmelink et al., 2016). When interpreting findings from both operant conditioning and non-operant conditioning paradigms, it is critical to consider that all these studies were performed using *mdx* mice on the C57BL/10ScSnJ genetic background. Thus, observations from these studies are specific to the genetic modifiers on the C57BL/10ScSnJ background.

Many neurophysiological processes critical to learning and memory are altered in mdx mice or indirectly affected by the absence of functional dystrophin including synaptic plasticity and cholinergic, GABAergic, and glutamatergic neurotransmission (Vaillend et al., 1999, 2004; Vaillend and Billard, 2002; Dallerac et al., 2011; Cohen et al., 2015; Vaillend and Chaussenot, 2017; Pereira da Silva et al., 2018). Effects on these mechanisms may underlie the behavioral alterations observed in the present study. Collectively, the behavioral and physiological studies suggest that: (1) *mdx* effects on learning may be task-specific, reward-specific, sex-specific, strain-specific, or specific to an interaction of some combination of these variables; and (2) these variables may interact with the *mdx* mutation to influence neurotransmitter networks and synaptic plasticity which ultimately influence behaviors including learning, memory, and visual discrimination.

### Stimulus Salience

Through its effect on bottom-up attentional processing, salience of visual stimuli plays a significant role in the ability to learn or express a response-reward relationship in humans (Cools et al., 2010), nonhuman primates (Crofts et al., 2001), and mice (Brigman and Rothblat, 2008; Dickson et al., 2013, 2014). In one of our previous studies (Dickson et al., 2014), we observed that stimulus-genotype interactions affecting stimulus salience may arise from genotype-dependent variation in the region of the stimulus to which mice attend, and that this phenomenon may be driven by subtle differences in body position, head position, or the visual field region to which mice attend. To address this in the present study, we developed visual stimuli that were equally bright and complex across all stimulus regions (Figure 1A). These stimuli were equally salient for both *mdx* mice and littermate controls (Figure 1B). This finding excludes the possibility that the facilitated visual discrimination in *mdx* mice observed in the present study (Figures 2A,B) was influenced by stimulus salience or an interaction of genotype and stimulus salience. Moreover, these data illustrate that the relative salience of visual stimuli *within* a dimension can be held constant by holding brightness and complexity constant across stimulus regions. This extends our previous finding that relative salience of visual stimuli *across* dimensions can be manipulated by manipulating stimulus characteristics for all stimuli within one of the stimulus dimensions (Dickson et al., 2014).

### Serial Reversal and Extinction Learning

Several studies have shown that executive function deficits are a component of the neurocognitive profile of DMD (reviewed in Snow et al., 2013). Cognitive flexibility, which can be indexed across species using the touchscreen reversal-learning task, is a core executive function enabling adaptation of learned behavior in the face of changing environmental demands (Dajani and Uddin, 2015). In the present study, serial reversal-learning performance was not affected by the *mdx* mutation (Figure 3). This is consistent with the work of Chaussenot et al. (2015) who found no effects of the *mdx* mutation on cognitive flexibility using a reversal-learning paradigm in the water maze and radial-arm maze. In contrast, Remmelink et al. (2016) observed a deficit in cognitive flexibility in female *mdx* mice in the absence of motor dysfunction or general learning impairments using the Barnes maze and a unique 3-choice assay that required crawling through one of three holes to obtain a food reward. In the present study, there was no effect of the *mdx* mutation on extinction learning (Figure 4). This observation is consistent with findings from a previous operant conditioning study in which extinction of a food reinforced operant bar press response was not affected by the *mdx* mutation (Vaillend and Ungerer, 1999). Collectively, these studies suggest that *mdx* effects on reversal learning may be task-specific or sex-specific; neither this study nor a previous study provides evidence supporting an effect of the *mdx* mutation on extinction learning. Observations from these studies are specific to the C57BL/10ScSnJ background and the genetic modifiers on that background.

### A Way Forward: Harnessing Genetic Complexity to Model Natural Variation in the Effects of Dystrophin Dysfunction on Cognitive Processes

Although the human literature indicates that cognitive deficits, including executive dysfunction, are a core component of the neurocognitive profile of DMD (reviewed in Snow et al., 2013), findings from the *mdx* mouse literature are much less consistent. One interpretation of these data is that the *mdx* mouse is a poor model of the cognitive and executive dysfunction that is observed in DMD. However, only ≈1/3 of individuals with DMD exhibit cognitive symptoms, and there is profound symptom heterogeneity in this population. This heterogeneity suggests that some individuals are genetically vulnerable to the effects of DMD perturbation on cognitive and executive functions, whereas others are genetically resistant to these effects. Thus, an alternative interpretation of the *mdx* mouse literature is that the C57BL/10ScSn background on which the *mdx* mutation is maintained confers only modest vulnerability to the effects of the *mdx* mutation on cognitive dysfunction. In this regard, the interaction of genetic background and single gene perturbation on cognitive phenotypes has been well described (e.g., Morice et al., 2004; Pietropaolo et al., 2011; Jaramillo et al., 2018). Differential effects of genetic background (C57BL/10ScSn or DBA/2J) on the noncognitive effects of the *mdx* mutation have been observed (Coley et al., 2016).

To fully understand the pathways and mechanisms through which dystrophin perturbation affects cognitive function, future work should focus on the ways in which specific cognitive phenotypes (e.g., discrimination learning, cognitive flexibility, working memory, reinstatement following extinction) are influenced, likely independently, by the interaction of *Dmd* perturbation and genetic background. This work should be done in both males and females and using multiple operant conditioning protocols (i.e., positive reinforcement, negative reinforcement, positive punishment, negative punishment). Recent advances in genetic engineering technologies (e.g., CRISPR) allow for rapid and relatively inexpensive genetic perturbation on multiple genetic backgrounds (Doench, 2018). An even more comprehensive approach was recently reported by Neuner et al. (2019) who developed an Alzheimer’s disease transgenic mouse reference panel by intercrossing a mouse model of Alzheimer’s disease with 28 strains from the BXD recombinant inbred panel. The interaction of the Alzheimer’s transgene and the 28 BXD genetic backgrounds resulted in broad phenotypic variation in cognitive function that mirrored the variation in human Alzheimer’s patients. By integrating cutting edge genetic engineering techniques with genetically complex mouse resources like the BXD or Collaborative Cross recombinant inbred panels (Dickson et al., 2015, 2016, 2018; Schoenrock et al., 2018; Bagley et al., 2019a,b), we can model natural variation in the effects of dystrophin dysfunction on cognition in order to disentangle the genetic mechanisms through which *Dmd* perturbation drives dysfunction in distinct cognitive processes.

## Data Availability

The datasets generated for this study are available on request to the corresponding author.

## Ethics Statement

### Animal Subjects

The animal study was reviewed and approved by IACUC at the University of Memphis.

## Author Contributions

PD and GM designed the experiments. PD performed the experiments, analyzed the data, and drafted the manuscript. Both authors contributed to finalize the manuscript for publication.

## Conflict of Interest Statement

The authors declare that the research was conducted in the absence of any commercial or financial relationships that could be construed as a potential conflict of interest.
